# Time-Lapse Imaging of Bismuth Precipitation and Coarsening on the Surface of Sn-Ag-Cu-Bi Solder Joints After Thermal Cycling

**DOI:** 10.1007/s11664-025-11747-4

**Published:** 2025-02-03

**Authors:** Chen-Lin Hsieh, Richard J. Coyle, Christopher M. Gourlay

**Affiliations:** 1 Department of Materials, Imperial College London, London, SW7 2AZ UK; 2 Nokia Bell Labs, Murray Hill, NJ USA

**Keywords:** Pb-free solder, microstructure, phase transformations, orientation relationships, thermal cycling

## Abstract

Adding bismuth to Sn-Ag-Cu solder compositions can significantly improve reliability in thermal cycling, but there are uncertainties in how bismuth precipitates and coarsens in Sn-Ag-Cu-Bi solders containing > 3 wt.% Bi. Here we apply time-lapse imaging in a scanning electron microscope to study bismuth precipitation and coarsening at room temperature on the polished surface of Sn-2.25-0.5Ag-6Bi ball grid array solder joints after thermal cycling. It is shown that (Bi) precipitates on the surface within 2 h after polishing and then coarsens by a combination of Ostwald ripening, coalescence ripening, and competition between two orientation relationships. Time-lapse imaging revealed that coalescence causes an increase in the local bismuth particle size and the formation of anomalously large (Bi) particles. The accumulation of bismuth on the polished surface increases far beyond the equilibrium volume fraction for this alloy. The bismuth particle size distributions are significantly wider than expected from Lifshitz–Slyozov–Wagner (LSW) theory, that assumes only Ostwald ripening, which is shown to be because coalescence creates anomalously large particles. This study shows the important role of bismuth precipitate coalescence within the coarsening mechanism in Sn-2.25-0.5Ag-6Bi solder joints.

## Introduction

Emerging applications for electronics in the automotive, aerospace, and defence sectors require solder joints to operate with high reliability under harsh environments. For these high-reliability applications, thermal fatigue of solder joints is a key reliability issue for surface-mount electronic components, which mostly results from the thermal expansion mismatch between electronic components and the printed circuit board.^1^

For the widely adopted Sn-Ag-Cu solders, eutectic Ag_3_Sn particles provide adequate particle strengthening in mild thermal cycling.^2,3^ However, under harsher thermal cycling, the strengthening effect from Ag_3_Sn particles diminishes, as Ag_3_Sn coarsens significantly at higher temperature and higher plastic strain.^1,4–6^ To improve thermal cycling reliability, a range of new Sn-Ag-Cu-based solders with additions of 1–7 wt.% of Sb, Bi, and/or In have been developed.^7–10^ Typically, the alloy design strategy is to make additions that provide (i) additional particle strengthening by (Bi) and/or SbSn precipitates^8,11,12^ and/or (ii) solid solution strengthening from the three elements in β-Sn which remains effective at high temperatures during thermal cycling.^12,13^

Among the three alloying elements, Bi is of particular interest, as it has relatively low cost and reduces the liquidus temperature^14,15^ as well as improving reliability. For example, 1 wt.% to 5 wt.% of Bi additions have been shown to enhance the shear strength,^14,16^ tensile strength,^14,17^ and creep resistance^15,18,19^ of Sn-Ag-Cu solders. More importantly, the thermal cycling reliability of Sn-Ag-Cu solder joints is usually enhanced by Bi additions.^8,20–22^ The improved mechanical properties have been attributed to (i) the solid solution strengthening by Bi in β-Sn, and (ii) the particle strengthening by Bi precipitates. Additionally, (Bi) additions improve the wettability,^14,23,24^ suppress the growth of interfacial Cu_3_Sn layers,^19,23,25^ and prevent the growth of tin whiskers^26,27^ in lead-free solders.

Previous studies have reported the presence of (Bi) phase that precipitated in β-Sn in Sn-3.8Ag-0.7Cu-4Bi,^28^ Sn-1.5Ag-0.7Cu-3Bi,^15^ and Sn-0.6Ag-0.7Cu-5Bi^29^ (wt.%) alloys at room temperature after solidification, whereas the same studies reported the absence of (Bi) phase in Sn-3.8Ag-0.7Cu-2Bi,^28^ Sn-1.5Ag-0.7Cu-1Bi,^15^ and Sn-0.6Ag-0.7Cu-2.5Bi^29^ (wt.%) alloys. From these studies, a Bi concentration of ≥ 3 wt.% seems to be required for (Bi) precipitates to form in β-Sn, although other factors may also be important. Different CALPHAD databases disagree on the solubility of Bi in β-Sn at 25°C,^30–32^ ranging from 2.1 wt.% in the National Institute of Standards and Technology (NIST) database^32^ up to 4.5 wt.% in the Thermo-Calc TCSLD4.1 database.^31^

At room temperature, Sn-Ag-Cu-Bi solders are at high homologous temperature (T/T_m_ ~ 0.6), which makes supersaturated β-Sn relatively unstable.^33^ Belyakov et al.^33^ investigated the precipitation and coarsening of (Bi) plates on the polished surface of Sn-3Ag-0.5Cu-4Bi solder balls, with faster kinetics than in the bulk. The precipitated (Bi) plates exhibited two crystallographic orientation relationships (ORs) with the β-Sn matrix^33^:

$$ \begin{aligned} & {\text{OR-1}}:\quad \{ {0 1 \overline{1} 2} \}_{{\left( {Bi} \right)}} \parallel \left\{ {1 0 0} \right\}_{\beta Sn} \& \langle2 \overline{2} 0 1\rangle_{{\left( {Bi} \right)}} \parallel \langle0 0 1\rangle_{\beta Sn} \\ & {\text{OR-2}}:\quad \left\{ {0 1 \overline{1} 2} \right\}_{{\left( {Bi} \right)}} \parallel \left\{ {1 0 0} \right\}_{\beta Sn} \& \langle2 \overline{2} 0 1\rangle_{{\left( {Bi} \right)}} \parallel \langle0 1 1\rangle_{\beta Sn} \\ \end{aligned}. $$

Under room-temperature ageing, the (Bi) precipitates with OR-1 coarsened at the expense of those with OR-2.^33^ On the other hand, (Bi) plates were found to thicken linearly with the cube root of time, indicating that the growth of (Bi) particles was controlled by volume diffusion in β-Sn.^33^

Wu et al.^34^ studied the precipitation and coarsening of Bi on the polished surface of Sn-3Ag-0.5Cu-3Bi solder joints. The removal of the surface also removed the precipitated (Bi) particles and reset the precipitation of (Bi), indicating the accumulation of (Bi) on the surface.^34^ During room-temperature ageing, the area of (Bi) particles increased linearly with the cube root of time, suggesting that the precipitation of (Bi) was controlled by the diffusion of Bi along the β-Sn grain boundary.^34^ Additionally, the higher cooling rate during soldering was found to retard the surface precipitation of (Bi).^34^

Although the above studies have provided clear evidence and analyses of the surface precipitation and coarsening of (Bi) particles, the understanding of detailed mechanisms is limited, especially the coarsening mechanisms of (Bi) particles on the surface. Furthermore, previous studies have focused on (Bi) precipitation in as-solidified solders, whereas the surface precipitation of (Bi) in thermally cycled solder joints remains unexplored. Thermal cycling should greatly change the morphology and distribution of (i) Bi solute in β-Sn and (ii) the (Bi) particles, as (Bi) repeatedly dissolves at the hot end of the thermal cycle and precipitates at the cold end during thermal cycling.^8,12,20^

In this work, we investigate the precipitation and coarsening of (Bi) particles on the surface of Sn-2.25Ag-0.5Cu-6Bi (wt.%) solder joints after thermal cycling using time-lapse imaging in a scanning electron microscope (SEM). The study focuses on two areas:

(i) Quantification of the coarsening behaviour of individual (Bi) particles and areas containing numerous (Bi) particles
(ii) Identification of the coarsening mechanisms at the scale of the individual (Bi) particles including the roles of coalescence ripening, Ostwald ripening, and crystallographic orientation relationships

It is important to note that the precipitation and coarsening of (Bi) on a free surface have been shown to be markedly different to bulk (Bi) precipitation and coarsening. For example, Belyakov et al.^33^ found that the kinetics of Bi precipitation and coarsening are more rapid on a polished surface than in the bulk, and Wu et al.^34^ reported the preferential accumulation of (Bi) at polished surfaces. Therefore, the results in this paper have significant differences to the behaviour of (Bi) phase within the bulk solder. However, a time-lapse study on the surface provides useful insights into the precipitation and coarsening mechanisms and the formation of preferred interfaces which are expected to occur in the bulk.

## Methods

Sn-2.25Ag-0.5Cu-6Bi (wt.%) solder balls, 460 μm in diameter, and paste of the same composition were used to solder a 192CABGA ball grid array (BGA) package to a printed circuit board (PCB) as described in detail by Coyle et al.^21^ The test vehicle was then thermally cycled between −55°C and 125°C for 4876 cycles. For each cycle, the cooling and heating rate was 10 K/min with a 10-min dwell time at the hot and cold ends of the cycle. After thermal cycling, a region of the PCB containing the package was cut out, mounted in Struers VersoCit-2 cold mounting resin, wet ground to 4000 grit SiC paper to near the centre of a row of joints, and then polished with colloidal silica.

To study subsequent microstructural evolution on the polished surface during room-temperature storage, the sample was characterised with time-lapse scanning electron microscopy (SEM). First the surface was imaged 2 h after polishing and the same areas were then characterised at multiple times until 2136 h after polishing. During the times between characterisation, the sample was stored at room temperature (~23°C). Analytical scanning electron microscopy was conducted on a Zeiss SIGMA field-emission gun SEM (Carl Zeiss, Oberkochen, Germany) with an Oxford Instruments INCA x-sight energy dispersive x-ray (EDX) detector (Oxford Instruments, Oxfordshire, UK) and a Bruker electron backscatter diffraction (EBSD) detector (Bruker AXS Inc., Fitchburg, WI, USA). Cross-sections were carbon-coated before SEM. Regions were also studied by EBSD and EDX mapping and were processed and analysed with Bruker Esprit 2.1 software and the MTEX toolbox in MATLAB.

**Fig. 1:**
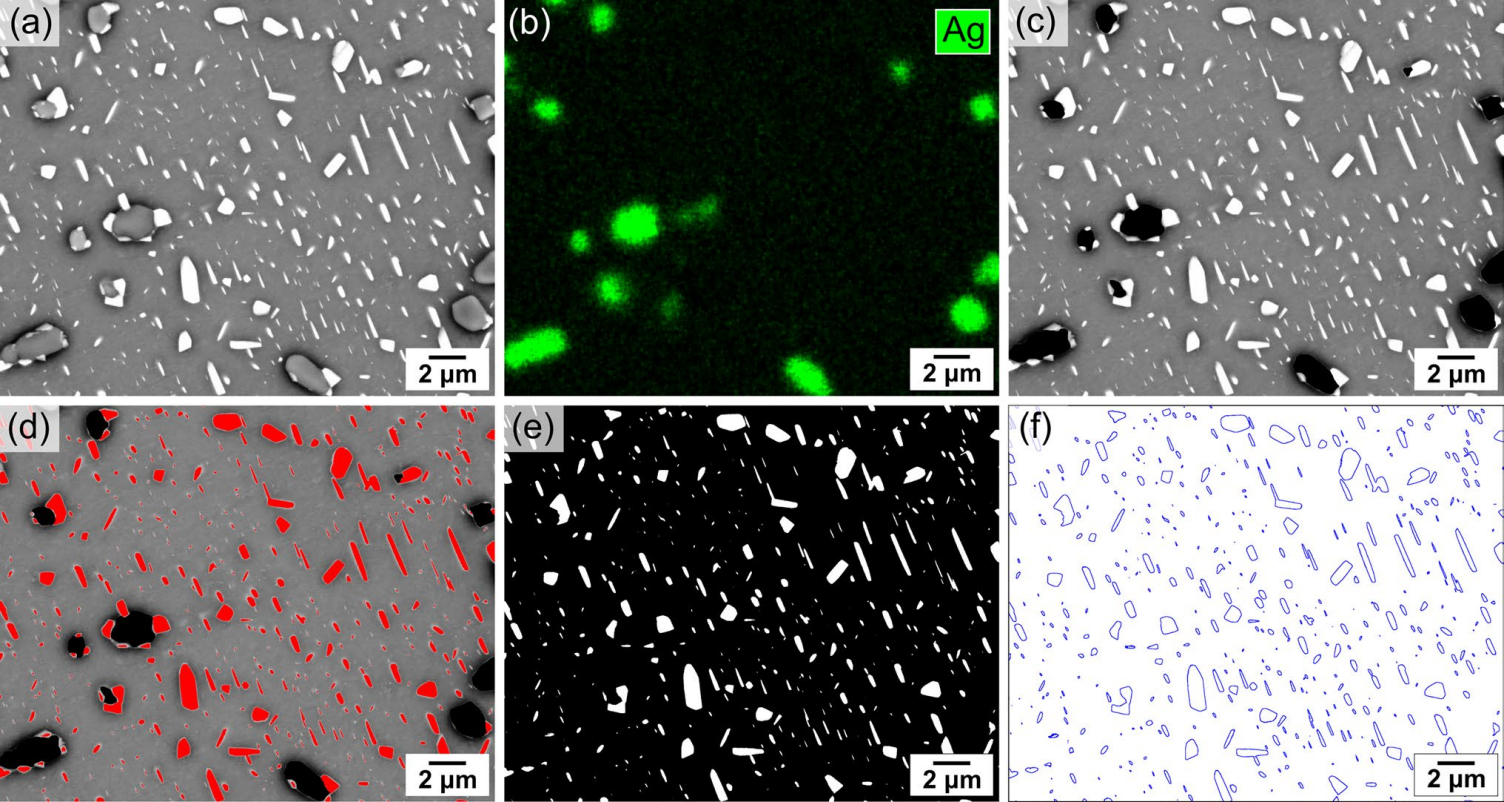
Image processing steps for analysing (Bi) particles in an area of a thermally cycled Sn-2.25Ag-0.5Cu-6Bi joint in the 192CABGA package. (a) BSE image. (b) Ag EDX maps of the area. (c) BSE image with Ag_3_Sn particles removed. (d) BSE image with (Bi) particles masked in red. (e) Binarized image of (Bi) particles. (f) Surface contours of (Bi) particles (Color figure online).

For time-lapse analysis, the time series of backscattered electron (BSE) images were aligned using Linear Stack Alignment with the Scale Invariant Feature Transform (SIFT) in ImageJ software.^35^ To analyse (Bi) particles, the aligned images were processed according to the method demonstrated in Fig. 1. Figure 1a shows a typical BSE image of a joint, with white particles being (Bi) phase and grey particles being Ag_3_Sn phase. Because of their brighter grayscale, the white (Bi) particles can be binarized using the threshold function of ImageJ. However, Ag_3_Sn particles could sometimes be mistakenly binarized due to the bright grayscale of small Ag_3_Sn and the centre of some Ag_3_Sn. To correctly segment the (Bi), all the Ag_3_Sn particles were identified by EDX mapping in Bruker Esprit 2.1 (Fig. 1b) and were then manually removed from the BSE image in ImageJ (Fig. 1c). Next, (Bi) particles were masked (Fig. 1d) and binarized using the threshold function in ImageJ (Fig. 1e). To further track the morphology evolution of individual (Bi) particles, the binarized (Bi) particles were outlined using the MATLAB Image Processing Toolbox (Fig. 1f). The binarized images and outline images were then used for particle size analysis.

## Results and Discussion

### Microstructural Evolution of Bi Particles During Room-Temperature Ageing

**Fig. 2:**
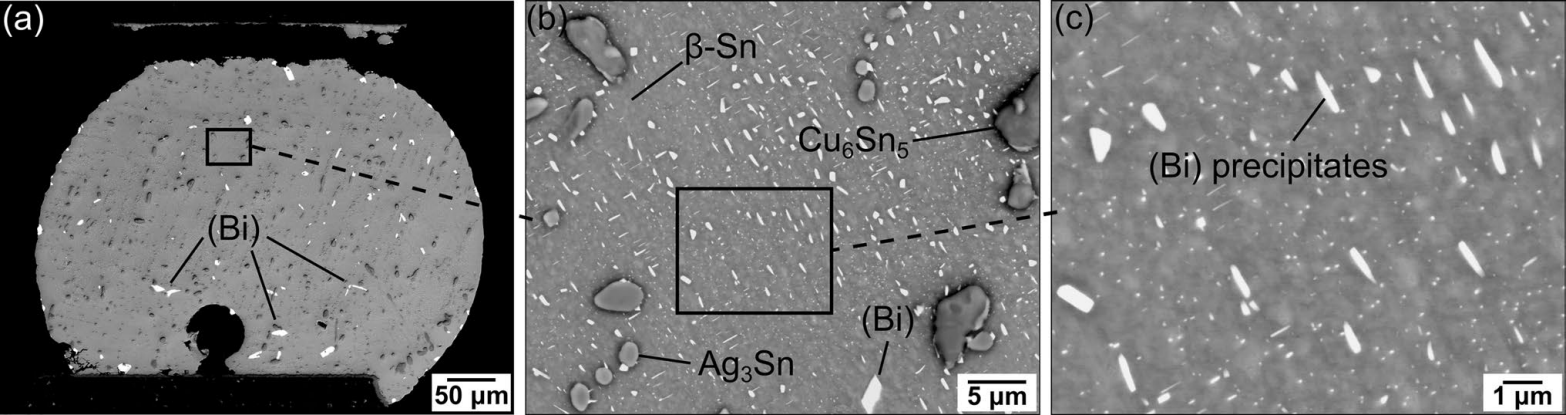
Microstructures of a thermally cycled Sn-2.25Ag-0.5Cu-6Bi solder joint in a 192CABGA package 73 h after polishing, showing (a) the whole joint, (b) the four common phases, and (c) surface-precipitated (Bi).

The BSE images in Fig. 2 display the typical microstructure of a thermally cycled Sn-2.25Ag-0.5Cu-6Bi solder joint surface 73 h after polishing. In Fig. 2a, some large, white (Bi) particles can be identified. These (Bi) particles precipitated inside the solder joint bulk, and their sizes ranged between 5 μm to 20 μm. Figure 2b shows the detailed microstructure and highlights the four phases present: β-Sn, Ag_3_Sn, Cu_6_Sn_5_, and (Bi). The original β-Sn dendrite structure is not discernible after thermal cycling.^1,4,5^ Additionally, there were no eutectic (Bi) particles, as they had dissolved and re-precipitated in β-Sn during thermal cycling. In the spaces between intermetallic particles, small (Bi) precipitates were uniformly distributed (Fig. 2c). Unlike the previously mentioned bulk-precipitated (Bi) particles, these small (Bi) particles only precipitated on the surface after the joint was polished. The following analyses will focus solely on these surface-precipitated (Bi) particles.

**Fig. 3:**
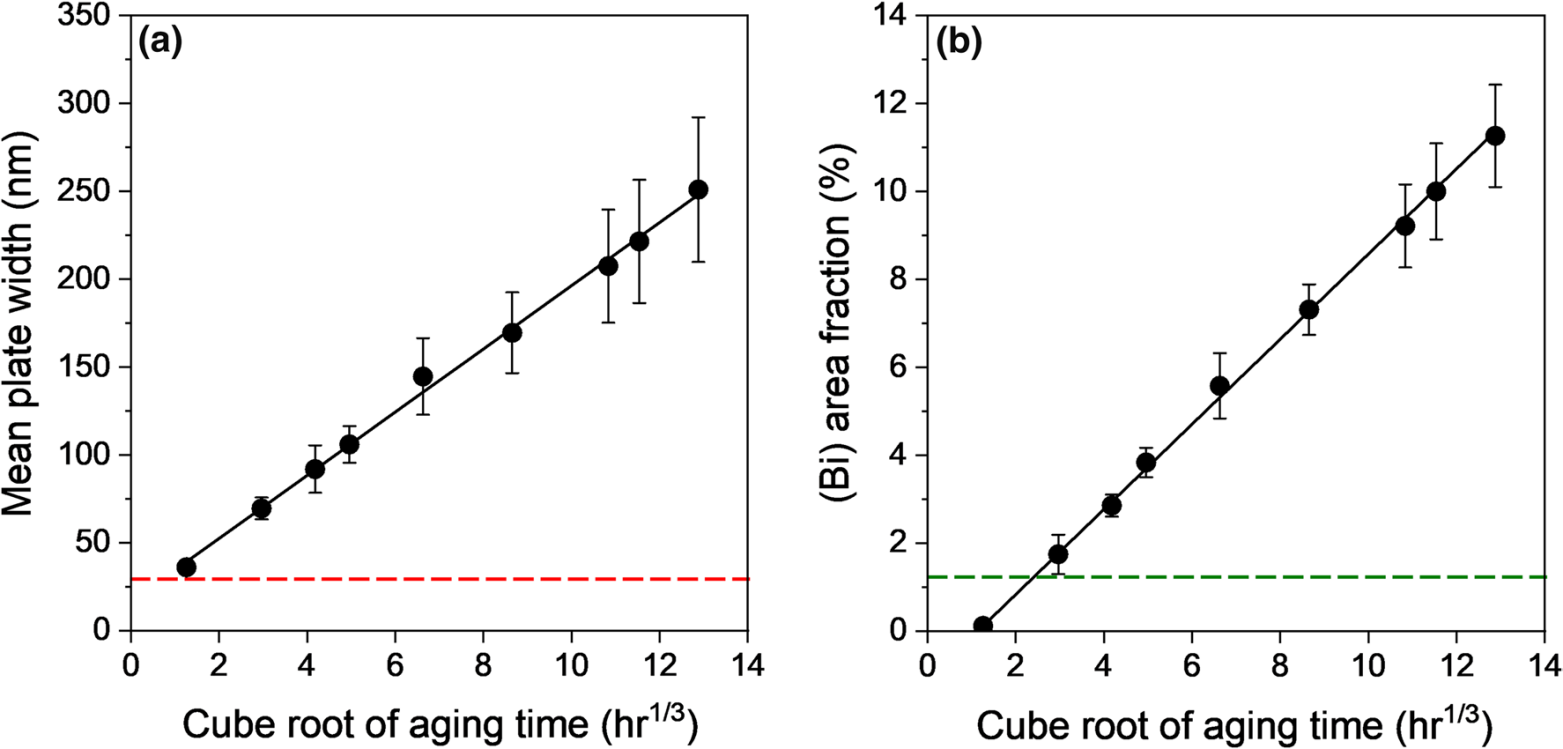
(a) Mean (Bi) plate width versus the cube root of ageing time. The red dashed line represents the resolution limit of the BSE images. (b) Area fraction of (Bi) phase on the sample surface versus the cube root of ageing time. The green dashed line represents the equilibrium volume fraction of (Bi) at 23°C (Color figure online).

Figure 3 shows the global precipitation and coarsening behaviour of at least 2500 (Bi) particles on the polished surface of three thermally cycled Sn-2.25Ag-0.5Cu-6Bi solder joints. Figure 3a and b are plots of the mean (Bi) plate width and the (Bi) phase area fraction versus the cube root of ageing time, respectively. The error bars in Fig. 3a and b represent the standard error of the three joints. The red dashed line in Fig. 3a represents the resolution limit of the BSE images, and the green dashed line in Fig. 3b represents the equilibrium volume fraction of (Bi) phase at 23°C for Sn-2.25Ag-0.5Cu-6Bi.

Both the mean plate width (Fig. 3a) and (Bi) area fraction (Fig. 3b) increased with time, showing that (Bi) was simultaneously coarsening and growing/accumulating on the surface during room-temperature ageing. The fitted lines in Fig. 3a and b indicate that both the mean plate width and (Bi) area fraction increased linearly with the cube root of time. In the case of the plate width in Fig. 3a, note that the measurement resolution limit (indicated by the red dashed line) is expected to cause smaller plates to be missed, especially at short times, which results in a positive intercept on the best-fit line. The linear relationship of mean (Bi) plate width with *t*^1/3^ is consistent with Belyakov et al.,^33^ and the linear relationship of (Bi) area fraction with *t*^1/3^ is consistent with Wu et al.,^34^ although they both studied samples after solidification whereas this study was performed after thermal cycling.

Figure 3b also shows that the (Bi) area fraction on the surface reached the equilibrium volume fraction at 23°C after 3 h^1/3^ (26 h) and continued increasing with time, reaching 11.3% area fraction by 12.9 h^1/3^ (~89 days). This indicates that (Bi) was preferentially accumulating at the surface during room-temperature ageing after polishing and confirms that surface phenomena are markedly different to bulk precipitation and coarsening in Sn-Ag-Cu-Bi alloys. Note also that the area fraction of (Bi) measured from above does not account for the protrusion of (Bi) above the β-Sn surface. Another feature of Fig. 3a and b is that the error bars indicate that the coarsening and growth rates of (Bi) particles vary across different solder joints and, locally, from area to area. This local behaviour is considered in detail next.

**Fig. 4:**
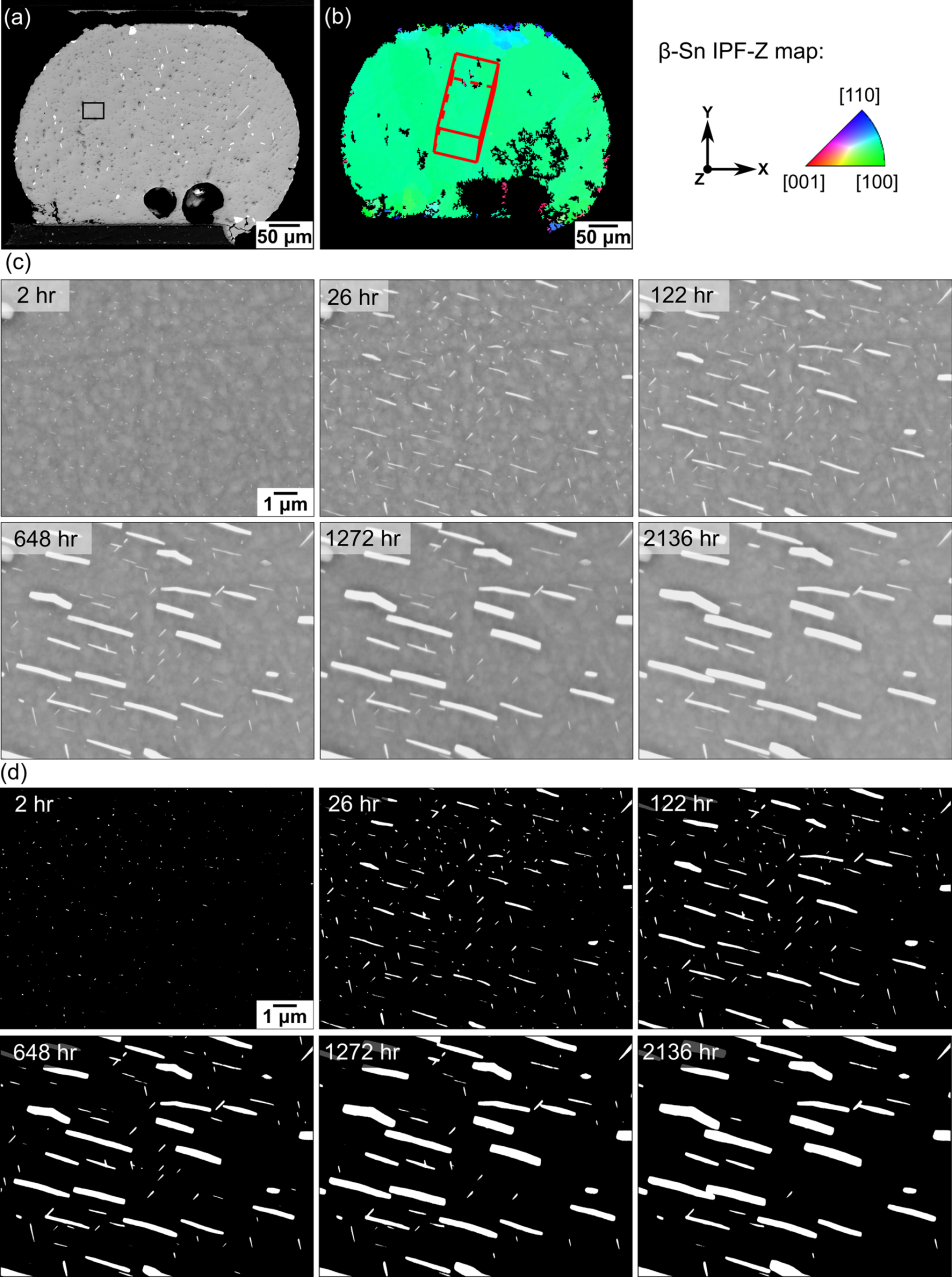
Overview of (Bi) precipitation and coarsening during 23°C ageing in a thermally cycled Sn-2.25Ag-0.5Cu-6Bi 192CABGA joint. (a) BSE image showing the location of the area in (c). (b) EBSD IPF-Z map superimposed with a β-Sn unit cell indicating the grain orientation of the joint. (c) Time-lapse BSE images and (d) time-lapse binarized images showing the evolution of (Bi) particles at six different times after polishing.

Figure 4 gives an example of the surface precipitation and coarsening of (Bi) on a single-grained Sn-2.25Ag-0.5Cu-6Bi joint in a thermally cycled 192CABGA package. The time-lapse BSE images in Fig. 4c show the evolution of (Bi) plates in the area highlighted in Fig. 4a from 2 h to 2136 h after the joint was polished. The binarized images in Fig. 4d provide a clearer view of the coarsening and significant increase in (Bi) area fraction, consistent with Fig. 3a and b.

The 2-h images in Fig. 4c and d show that (Bi) particles had precipitated within 2 h after polishing. These particles were smaller than 30 nm and close to the resolution limit of 14 nm, so only a portion of these particles were binarized and shown in Fig. 4d. It was found that all (Bi) particles had precipitated out within 2 h, and no new (Bi) particles were observed to precipitate afterwards.

Figure 4c and d also show that most of the (Bi) particles displayed a plate-like morphology, with larger (Bi) plates aligned with the [0 0 1]_*βSn*_ direction of the β-Sn matrix (compare Fig. 4c and d with the unit cell wireframe in Fig. 4b), and some smaller (Bi) plates were aligned in two minor directions (Fig. 4c and d). It can be seen that, from 26 h onwards, the (Bi) plates aligned with [0 0 1]_*βSn*_ became dominant, while the other (Bi) plates shrank and mostly dissolved.

**Fig. 5:**
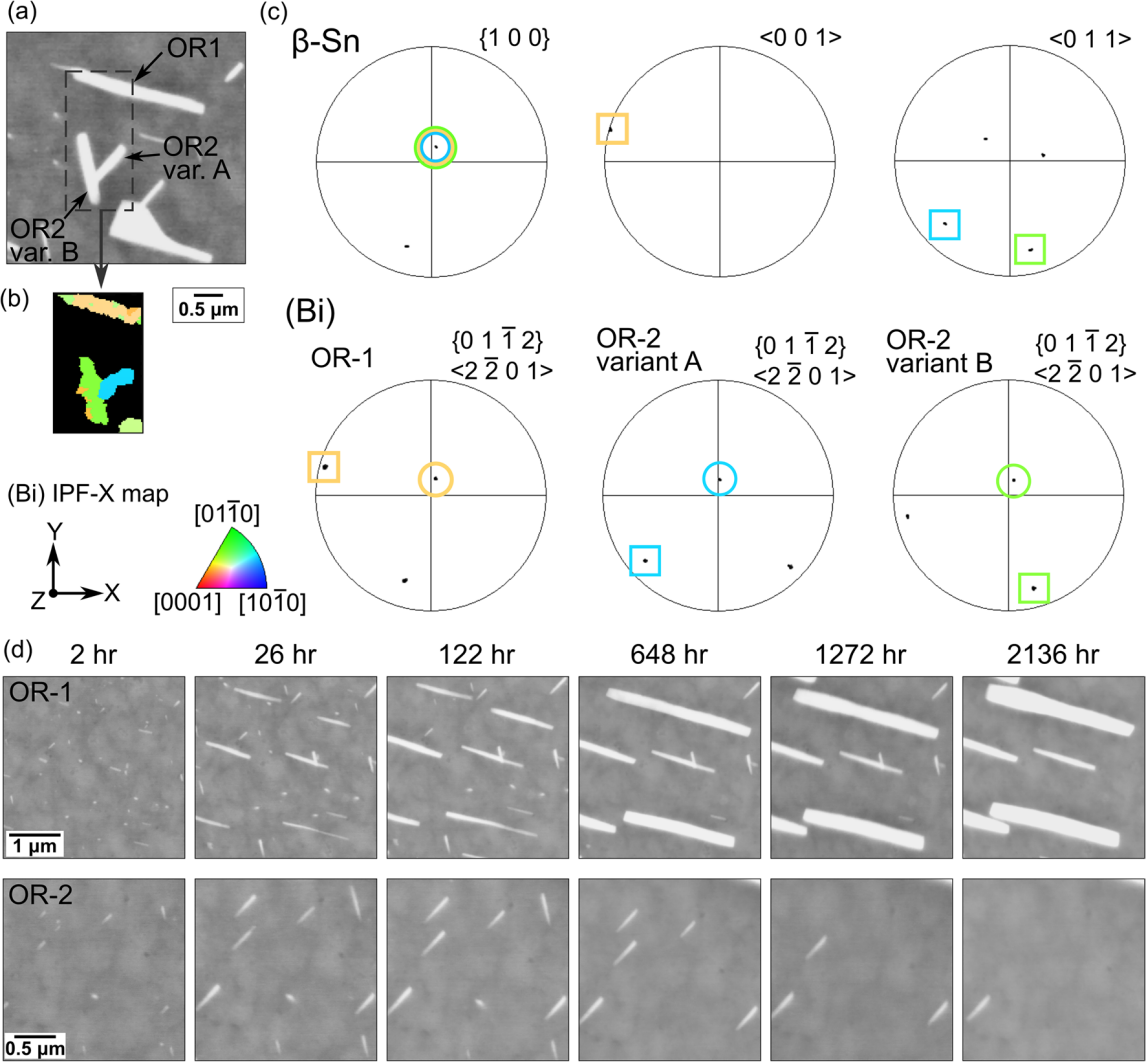
(a) BSE image, (b) EBSD IPF-X map of (Bi) in the boxed area in (a), and (c) EBSD pole figures of the three (Bi) plates and β-Sn matrix in (a) which have OR-1 or OR-2. N.B. the (Bi) pole figures are plotted for {$01\overline{1}2$} which are almost collinear with the <$2\overline{2}01$>. (d) Time-lapse BSE images showing (Bi) plates with OR-1 (top) and OR-2 (bottom) to β-Sn at six times after polishing.

The crystallographic orientation of (Bi) plates aligned along different directions was further analysed by EBSD as shown in Fig. 5. Results from a region containing both types of plate alignment are given in Fig. 5a–c. The pole figures in Fig. 5c show that the (Bi) plates aligned with [0 0 1]_*βSn*_ shared OR-1 with the β-Sn matrix, while those aligned in the minor directions shared OR-2 with the β-Sn matrix, which is consistent with the findings by Belyakov et al.^33^:

$$ \begin{aligned} & {\text{OR-1:}}\quad \{ {0 1 \overline{1} 2} \}_{{\left( {Bi} \right)}} \parallel \left\{ {1 0 0} \right\}_{\beta Sn} \& \langle2 \overline{2} 0 1\rangle_{{\left( {Bi} \right)}} \parallel \langle0 0 1\rangle_{\beta Sn} \\ & {\text{OR-2:}}\quad \left\{ {0 1 \overline{1} 2} \right\}_{{\left( {Bi} \right)}} \parallel \left\{ {1 0 0} \right\}_{\beta Sn} \& \langle2 \overline{2} 0 1\rangle_{{\left( {Bi} \right)}} \parallel \langle0 1 1\rangle_{\beta Sn} \\ \end{aligned}. $$

The top row and the bottom row of Fig. 5d display time-lapse BSE images of (Bi) plates with OR-1 and those with OR-2, respectively. Note that the two rows of BSE images are presented at different magnifications for better visibility. The (Bi) plates with OR-1 continued growing over time (Fig. 5d). After 648 h, the (Bi) plates stopped lengthening but continued to thicken. In contrast, as shown in the second row of Fig. 5d, the (Bi) plates with OR-2 only grew in the first 26 h and gradually dissolved afterwards. In Belyakov et al.,^33^ the preferred growth of (Bi) plates with OR-1 was attributed to the better coherency at the $\{ {0 1 \overline{1} 2} \}_{{\left( {Bi} \right)}} \langle2 \overline{2} 0 1\rangle_{{\left( {Bi} \right)}} \parallel \left\{ {1 0 0} \right\}_{\beta Sn} \langle0 0 1\rangle_{\beta Sn}$ interface of OR-1 compared to the $\{ {0 1 \overline{1} 2} \}_{{\left( {Bi} \right)}} \langle2 \overline{2} 0 1\rangle_{{\left( {Bi} \right)}} \parallel \left\{ {1 0 0} \right\}_{\beta Sn} \langle0 1 1\rangle_{\beta Sn}$ interface of OR-2. Due to the lower interfacial coherency, the (Bi) plates with OR-2 likely have higher interfacial energy than OR-1 in addition to being smaller than plates with OR-1. Both of these factors will promote the growth of large particles with OR-1 and the dissolution of small plates with OR-2 during Ostwald ripening.

**Fig. 6:**
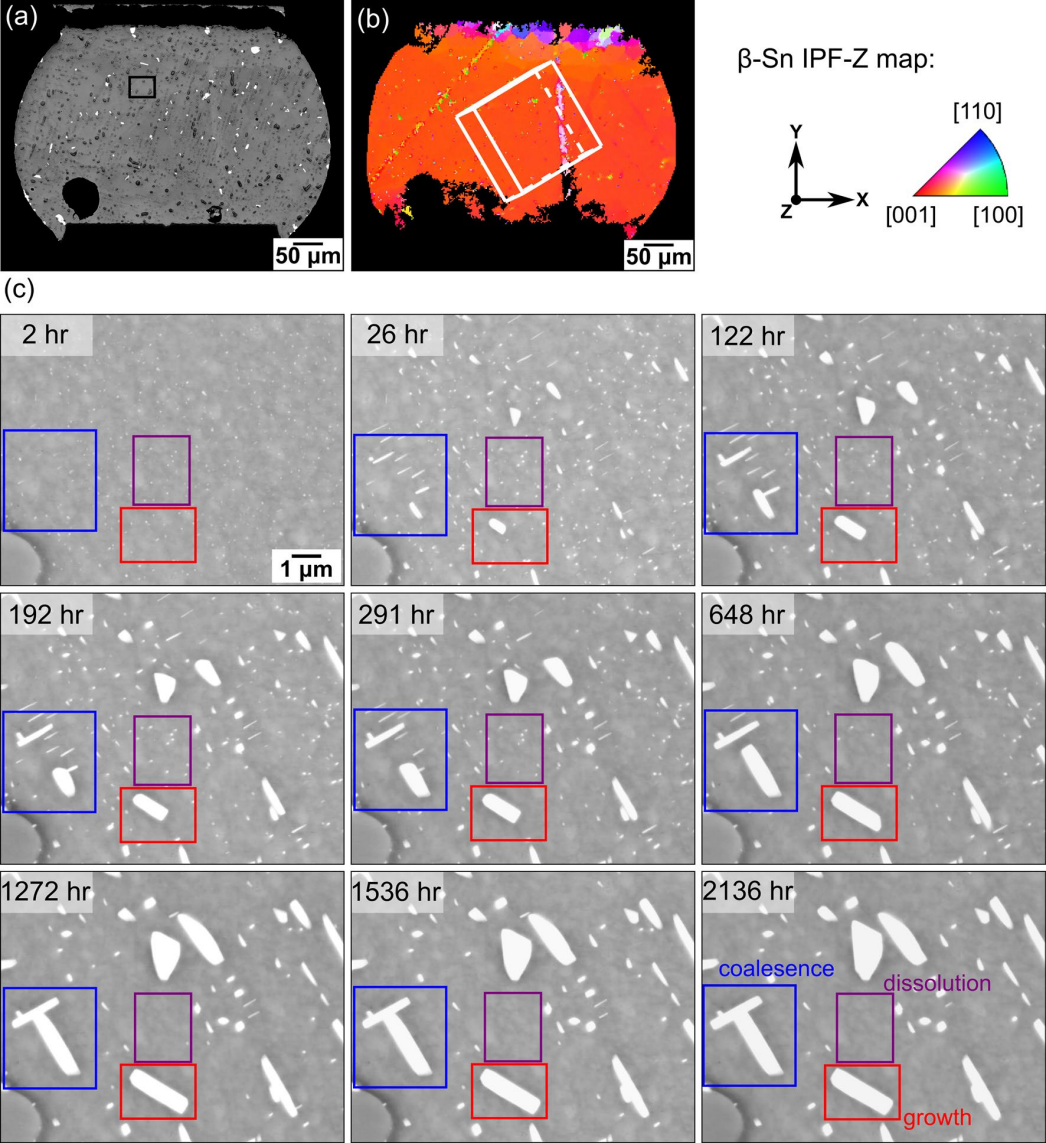
Microstructural evolution of a thermally cycled Sn-2.25Ag-0.5Cu-6Bi 192CABGA joint aged at 23°C. (a) BSE image indicating the location of (c) in the joint. (b) EBSD IPF-Z map showing the β-Sn grain orientation of the joint. (c) Time-lapse BSE images of an area. The boxes show particles undergoing growth (red), coalescence (blue), and dissolution (purple) (Color figure online).

**Fig. 7:**
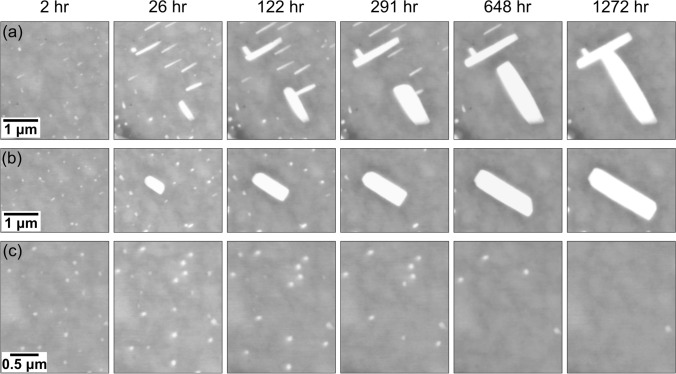
Time-lapse BSE images showing the three phenomena of (Bi) particle coarsening highlighted in Fig. 6. (a) Coalescence, (b) growth, and (c) dissolution.

The coarsening mechanisms of (Bi) particles on the surface are displayed in Fig. 6 and Fig. 7 using another example of a single-grained Sn-2.25Ag-0.5Cu-6Bi solder joint in a thermally cycled 192CABGA package. Note in Fig. 6 that the (Bi) plates here were less aligned than in Fig. 5 because the [0 0 1]_*βSn*_ direction is nearly normal to the cross-section in Fig. 6b. Figure 6c shows the evolution of (Bi) particles in the area highlighted in Fig. 6a from 2 h to 2136 h after polishing. The blue boxes in Fig. 6c show the coalescence ripening of (Bi) particles, where two (Bi) plates continuously grew and merged with other smaller (Bi) plates, eventually forming an anomalously large (Bi) particle. The red boxes and purple boxes in Fig. 6c show Ostwald ripening of (Bi) particles, where the (Bi) particle in the red boxes grew and the Bi particles in purple boxes dissolved.

The boxed areas in Fig. 6c are shown in more detail in Fig. 7. The top and middle rows are presented at the same magnification, but the bottom row is presented in a higher magnification for better visibility. Figure 7a highlights the coalescence process of (Bi) particles marked by the blue boxes in Fig. 6c. At 122 h, a (Bi) plate at the bottom right corner coalesced with a smaller (Bi) particle, and a (Bi) plate at the top left corner coalesced with another smaller (Bi) particle (Fig. 7a). At 1272 h, the two coalesced (Bi) particles further coalesced with each other, which resulted in an increase in the particle size. Note that the particle coarsening in Fig. 7a was also facilitated by Ostwald ripening, where the large (Bi) particle at the bottom right corner grew rapidly between 122 h and 648 h at the expense of the three dissolving (Bi) plates in the middle area.

Figure 7b and c highlight the particle growth and dissolution in the Ostwald ripening of (Bi) particles, corresponding to the red and purple boxes in Fig. 6c, respectively. The (Bi) particle in Fig. 7b grew continuously from 2 h to 1272 h without merging with any other (Bi) particle. In contrast, the (Bi) precipitates in Fig. 7c grew to their maximum size at 26 h, and then gradually dissolved thereafter. Comparing the (Bi) particles in Fig. 7a with the particle in Fig. 7b, the largest particle in Fig. 7a was initially smaller at 26 h than the largest particle in Fig. 7b, but became larger after coalescence at the end. This demonstrates how coalescence ripening enhances the coarsening of (Bi) particles compared to Ostwald ripening alone and shows how coalescence can create anomalously large particles.

### Quantification of Time-Lapse Images and Individual Particles

**Fig. 8:**
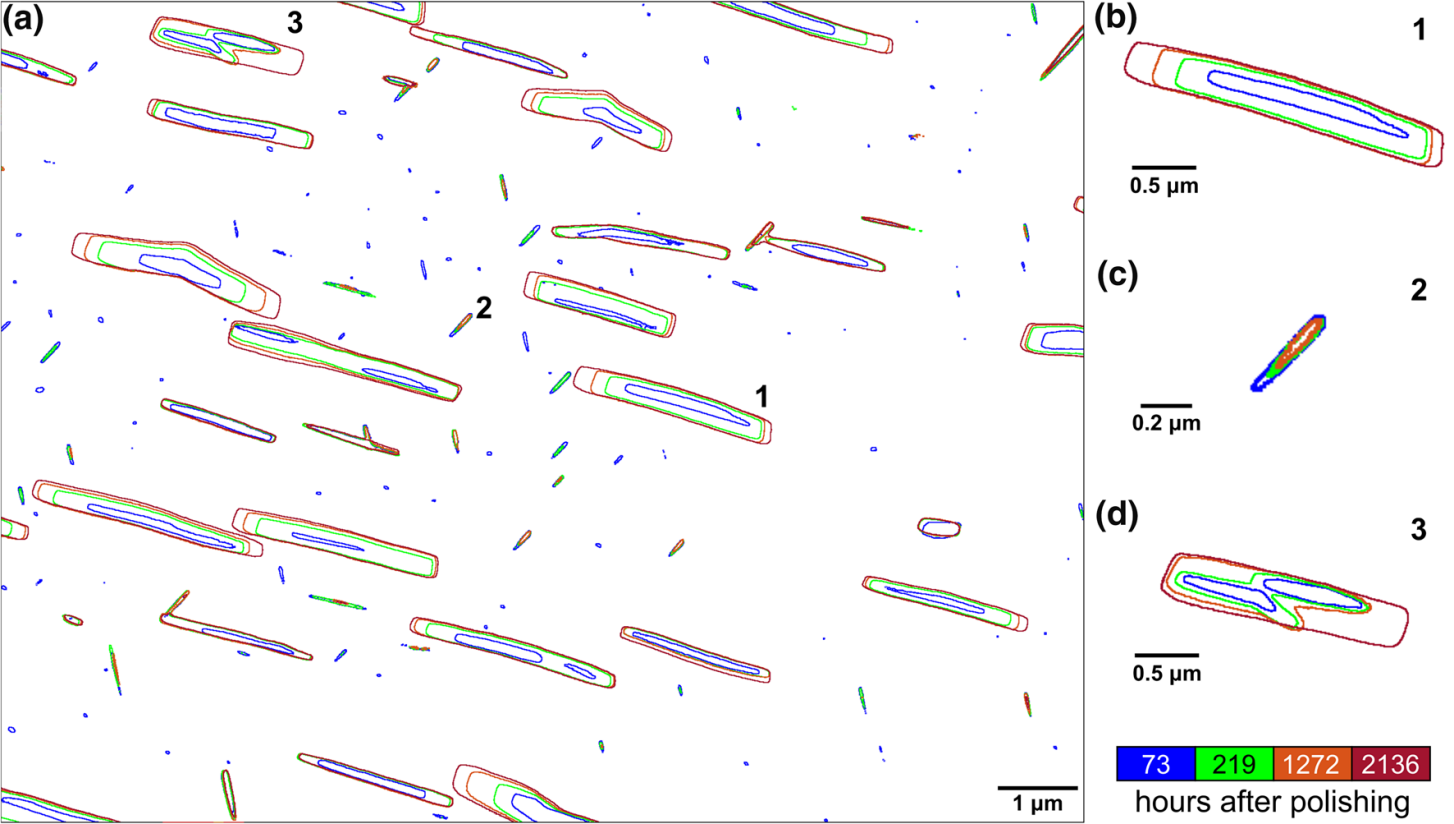
Interface contours of (Bi) particles coloured according to different times after polishing. (a) The whole area. (b, c, d) Individual (Bi) particles. (b) Particle 1 grew. (c) Particle 2 shrank. (d) Particle 3 formed by coalescence from two (Bi) plates.

Figure 8 displays the morphological change in individual (Bi) particles in the same area as Fig. 4c from 73 h to 2136 h after polishing. Using the method shown in Fig. 1, the interface contour of each (Bi) particle was identified and assigned a colour based on the time the BSE image was taken, as indicated by the colour legend below (Fig. 8a). Examples of the evolution of (Bi) plates with OR-1 and OR-2 to β-Sn are given in Fig. 8b and c, respectively. In Fig. 8b, the (Bi) plate with OR-1 grew continuously from the blue contour at 73 h to the red contour at 2136 h. In contrast, in Fig. 8c, the (Bi) plate with OR-2 shrank from the blue contour at 73 h to the orange contour at 1272 h, and eventually disappeared at 2136 h.

Figure 8d further shows the morphology change during the coalescence of two (Bi) plates. As shown by the green contour in Fig. 8d, the two (Bi) plates coalesced at 219 h, forming an irregular particle where the shapes of the two initial (Bi) plates were still distinguishable. At 1272 and 2136 h, the concavities of the coalesced particle were gradually filled, resulting in a more rectangular morphology (Fig. 8d).

The (Bi) plates in both Fig. 8b and d developed straighter (Bi)+β-Sn interfaces during coarsening. Based on the EBSD analysis in Fig. 4, the longer interfaces of the rectangular Bi plates correspond to the $\{ {0 1 \overline{1} 2} \}_{{\left( {Bi} \right)}} \langle2 \overline{2} 0 1\rangle_{{\left( {Bi} \right)}} \parallel \left\{ {1 0 0} \right\}_{\beta Sn} \langle0 0 1\rangle_{\beta Sn}$, which has the highest coherency.^33^ The shorter interfaces correspond to $\{ {0 1 \overline{1} 2} \}_{{\left( {Bi} \right)}} \langle2 \overline{2} 0 1\rangle_{{\left( {Bi} \right)}} \parallel \left\{ {0 0 1} \right\}_{\beta Sn}\langle 1 0 0\rangle_{\beta Sn}$, which are less coherent than the longer interface but more coherent than other possible interfaces. Thus, the development of straight interfaces with these planes reduces the total interfacial energy by both reducing the interfacial energy per unit area and reducing the interfacial area.^36^

**Fig. 9:**
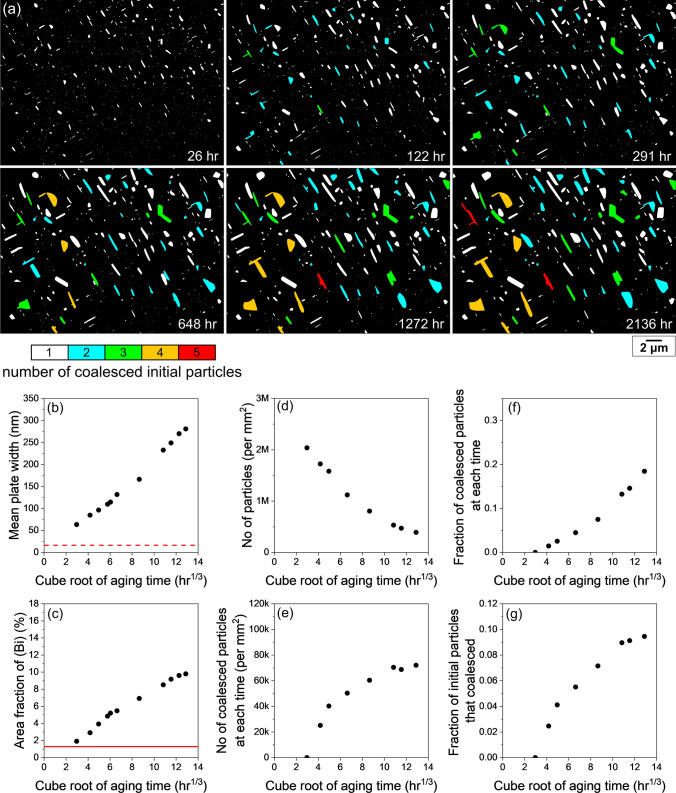
Quantification of (Bi) particle coalescence. (a) Time-lapse colour maps showing number of coalesced initial (Bi) particles in each (Bi) particle at different ageing time. (b) Mean (Bi) plate width at each ageing time. The dashed red line is the resolution limit. (c) Area fraction of (Bi) at each ageing time. The solid red line is the equilibrium volume fraction of (Bi) for Sn-2.25Ag-0.5Cu-6Bi at 23°C. (d) The total number of particles per mm^2^ at each ageing time. (e) Number of coalesced particles per mm^2^ at each ageing time. (f) Fraction of coalesced particles at each ageing time. (g) Fraction of initial particles that become coalesced at each ageing time (Color figure online).

Figure 9 presents the quantification of coalescence in a larger area that includes the area of Fig. 6. Figure 9a shows colour maps of (Bi) particles at six times, where each particle was coloured based on the number of initial particles that the particle was coalesced from. Initial particles here are defined as the (Bi) particles in the BSE image 26 h after polishing. The (Bi) particles at 2 h were excluded from these quantifications since many Bi particles are smaller than the resolution limit of BSE images at this time. As shown in Fig. 9a, 18.4% of (Bi) particles had coalesced after 2136 h, and two particles, coloured in red, were coalesced from five initial particles.

Figure 9b and c show that the increases in plate width and area fraction of (Bi) scale approximately with the cube root of time in this local area, which is consistent with the global behaviour in Fig. 3. In Fig. 9d, the number density of (Bi) particles in this area drops by more than a factor of 5, indicating a significant coarsening of (Bi) particles.

Figure 9e presents the number density of coalesced particles at each time. Here, coalesced particles are defined as the particles containing multiple particles that merged during coarsening, i.e., the coloured particles in Fig. 9a. The number density increased rapidly at the first four times, and then slowed and reached a plateau value of ~70,000 per mm^2^ at the end (Fig. 9e). Note that the slight decrease in the number density between 10.8 h^1/3^ and 11.5 h^1/3^ (Fig. 9e) was caused by the coalescence between already coalesced particles.

Figure 9f is a plot of the fraction of existing coalesced (Bi) particles over the total number of (Bi) particles at each time, which was obtained by dividing the values in Fig. 9e by the values in Fig. 9d. The fraction increased near-linearly with the cube root of time and reached 18.4% at the end (Fig. 9f). In other words, in the 2136-h colour map of Fig. 9a, the coloured particles account for 18.4% of the total particles.

Figure 9g shows the fraction of initially precipitated (Bi) particles that had coalesced at each time. This fraction increased over time and finally reached 9.5%, meaning 9.5% of particles present at 26 h went on to coalesce with other (Bi) particles during the subsequent 2136 h of room-temperature aging.

**Fig. 10:**
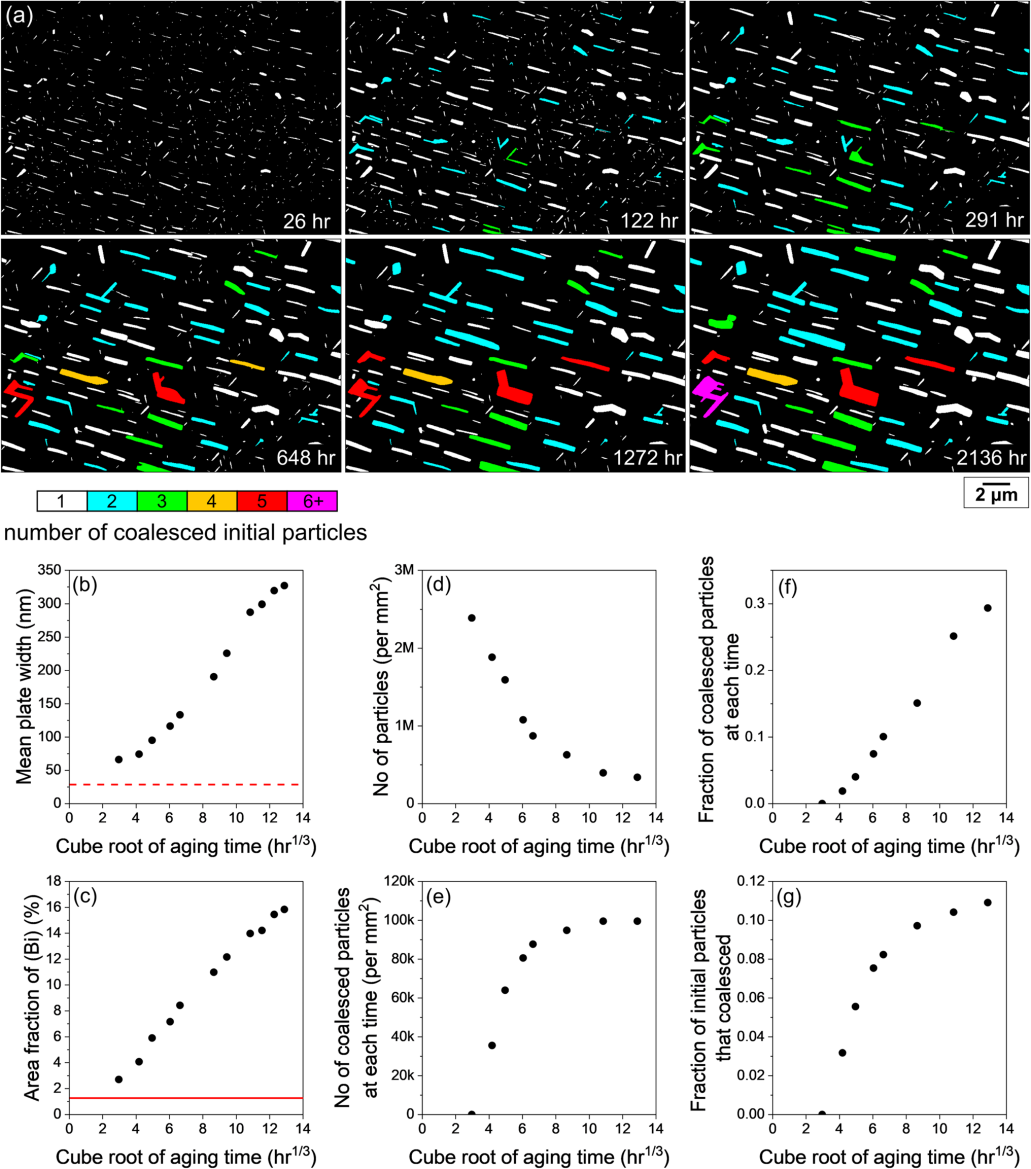
Quantification of (Bi) particle coalescence. (a) Time-lapse colour maps showing number of coalesced initial (Bi) particles in each (Bi) particle at different ageing time. (b) Mean (Bi) plate width at each ageing time. The dashed red line is the resolution limit. (c) Area fraction of (Bi) at each ageing time. The solid red line is the equilibrium volume fraction of (Bi) for Sn-2.25Ag-0.5Cu-6Bi at 23°C. (d) The total number of particles per mm^2^ at each ageing time. (e) Number of coalesced particles per mm^2^ at each ageing time. (f) Fraction of coalesced particles at each ageing time. (g) Fraction of initial particles that become coalesced at each ageing time (Color figure online).

To give an indication of the variability in coarsening behaviour from region to region, another example is given in Fig. 10 which is from an area close to the area in Fig. 4. The colour scale in Fig. 10a is the same as that in Fig. 9a, and the axis limits in Fig. 10b–g are the same as those in Fig. 9b–g. While the general behaviour and the shape of the plotted data are similar in these areas of microstructure in Fig. 9 and Fig. 10, the kinetics of (Bi) accumulation on the surface, coarsening, and coalescence are all significantly faster in the area in Fig. 10. For example, comparing Fig. 9 and Fig. 10, we see in Fig. 10 that the area fraction of (Bi) increased more rapidly (panel c), the coarsening rate was faster (the slope in panel b), and coalescence was more extensive (panels d–g). Specifically, in Fig. 10, there is a steeper rise in the initial number of coalesced particles with time (e), a higher fraction of coalesced particles at any given time (f), and a higher final fraction of initial particles that coalesced (g). This can be further seen in panel (a), where the maximum number of initial particles that coalesced was five in Fig. 9a and eight in Fig. 10a. The plateau shapes in Fig. 10e and g are flatter than in Fig. 9e and g, seemingly because when more particles have coalesced, there is less possibility for continued coalescence.

**Fig. 11:**
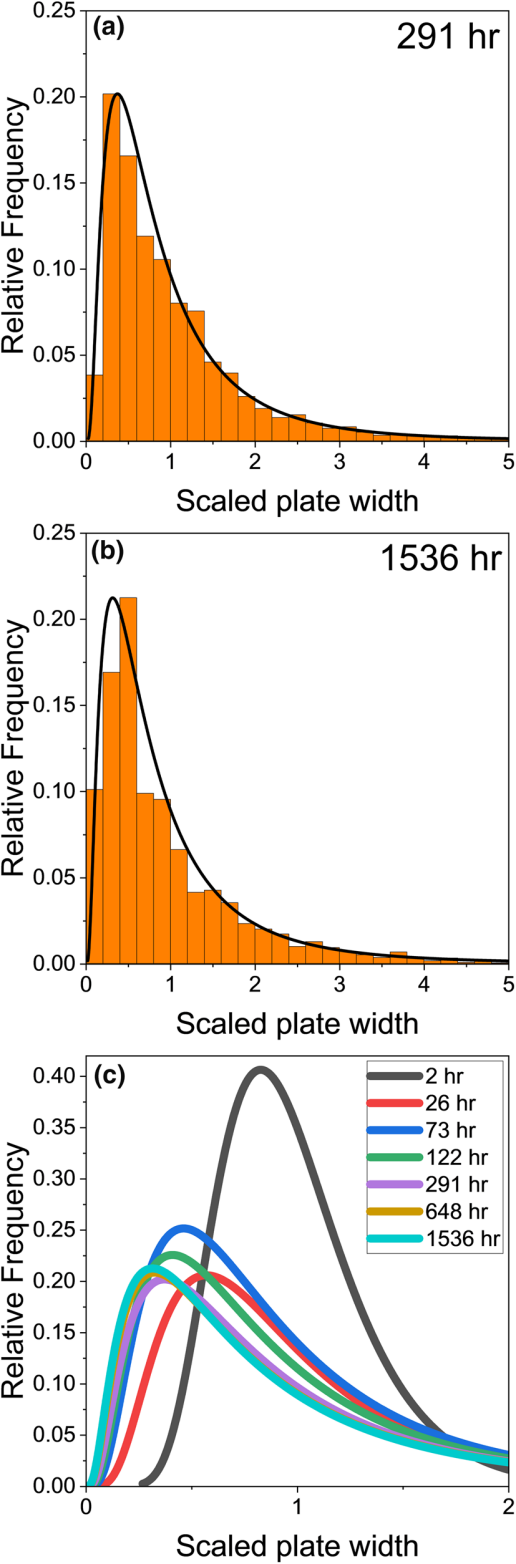
Histograms showing the scaled plate width distributions of (Bi) particles (a) 291 h and (b) 1536 h after polishing. (c) Fitted log-normal curves of the scaled plate width distributions at seven times.

To gain further insights into the coarsening mechanisms, Fig. 11 shows the scaled plate width distributions of (Bi) particles at different times after polishing. The scaled width is defined as the plate width divided by the mean plate width at each time in each area. For each time, at least 2500 (Bi) particles were measured from BSE images across three joints. Figure 11a and b show the distributions at 291 h and at 1536 h, which have similar shapes and are both reasonably well fit by log-normal distributions. Both distributions are skewed to smaller scaled plate width and have tails at large plate width that extend to five scaled plate widths. Note that for an Ostwald ripening mechanism, Lifshitz–Slyozov–Wagner (LSW) theory^37^ predicts that the scaled plate width distribution should not exceed 1.5. The extension of the distribution to ~5 is due to the presence of a few anomalously large (Bi) particles caused by the coalescence of (Bi) particles shown in detail in Fig. 9 and Fig. 10. That is to say, the scaled plate width distribution is due to both Ostwald ripening and coalescence ripening phenomena.

Figure 11c combines the fitted log-normal distributions at seven times. At 2 h, the distribution was skewed slightly to smaller scaled plate width and shows a peak at 0.8 scaled plate width (Fig. 11c). At 26 h, in contrast, the distribution was skewed significantly to smaller scaled plate width, with the peak close to 0.5 (Fig. 11c). The difference is probably because, at 2 h, the distribution is mostly due to precipitation, whereas at 26 h, the distribution is dominated by coarsening phenomena. One factor is that the anomalously large coalesced particles increase the mean plate width, thereby skewing the distribution at 26 h to smaller scaled plate width.

From 26 h onwards, the distributions are approximately time-independent and self-similar, which is common to both Ostwald ripening (LSW) theory^37^ and coalescence ripening theory,^38^ and is consistent with the result of Belyakov et al.^33^ However, the shapes of the scaled plate width distributions differ from those predicted by LSW theory^37^ and coalescence ripening,^38^ which may be related to surface precipitation and possibly because the area fraction of (Bi) particles on the surface increased with time (Fig. 3).

## Conclusions

This study has revealed new insights into the stability of the (Bi) phase in Sn-2.25Ag-0.5Cu-6Bi BGA solder joints by performing time-lapse imaging on polished surfaces after thermal cycling. From quantification of the precipitation, coarsening, and accumulation of (Bi) phase on the surface, the following conclusions can be drawn:

(Bi) particles generally precipitated on the surface within 2 h after polishing, and no new (Bi) particles were observed thereafter.
The coarsening of (Bi) on the surface was governed by Ostwald ripening, coalescence ripening, and the increasing area fraction of (Bi) accumulating on the surface, which reached an area fraction more than seven times the equilibrium volume fraction of (Bi) in this composition at 23°C.
During 2136 h of room-temperature ageing, up to eight (Bi) particles coalesced together, resulting in the increase in particle size and the formation of anomalously large (Bi) particles.
(Bi) particles preferentially developed straight $\{ {0 1 \overline{1} 2} \}_{{\left( {Bi} \right)}} \langle2 \overline{2} 0 1\rangle_{{\left( {Bi} \right)}} \parallel \left\{ {1 0 0} \right\}_{\beta Sn} \langle0 0 1\rangle_{\beta Sn}$ and $\{ {0 1 \overline{1} 2} \}_{{\left( {Bi} \right)}}\langle 2 \overline{2} 0 1\rangle_{{\left( {Bi} \right)}} \parallel \left\{ {0 0 1} \right\}_{\beta Sn}\langle 1 0 0\rangle_{\beta Sn}$ interfaces during particle coarsening.
The coalescence of (Bi) particles was quantified in two areas. For both areas, approximately 10% of (Bi) particles present at 26 h went on to coalesce with other (Bi) particles during the subsequent 2136 h of room-temperature ageing.
The extent of coalescence varied from region to region. Coalescence was more extensive in regions with faster (Bi) precipitation and coarsening.
From 26 h onwards, the scaled plate width distributions of (Bi) particles were skewed to smaller scaled plate width and had tails at large plate width that extended to five scaled plate widths. This distribution was caused by the anomalously large (Bi) particles formed through particle coalescence.
